# Extracorporeal Membrane Oxygenation for COVID-19-Associated Multisystem Inflammatory Syndrome in a 5-year-old

**DOI:** 10.1177/0003134820983198

**Published:** 2022-02

**Authors:** Stephanie P. Schwartz, Tracie C. Walker, Margaret Kihlstrom, Mubina Isani, Melissa M. Smith, Rebecca L. Smith, Sean E. McLean, Katherine C. Clement, Michael R. Phillips

**Affiliations:** 1 Division of Pediatric Critical Care Medicine, Department of Pediatrics, 549964 University of North Carolina, Chapel Hill, NC, USA; 2 Division of Pediatric Surgery, Department of Surgery, 214495 University of North Carolina, Chapel Hill, NC, USA

**Keywords:** extracorporeal membrane oxygenation, COVID-19, multisystem inflammatory syndrome in children, pediatrics, critical care

## Abstract

Severe acute respiratory distress syndrome coronavirus 2 (SARS-CoV-2) is associated with multisystem inflammatory syndrome in children (MIS-C) that ranges from mild symptoms to cardiopulmonary collapse. A 5-year-old girl presented with shock and a rapid decline in left ventricular function requiring intubation. SARS-CoV-2 was diagnosed by viral Polymerase Chain Reaction (PCR), and she received remdesivir and COVID-19 convalescent plasma. Initial echocardiogram (ECHO) demonstrated low normal left ventricular function and mild left anterior descending coronary artery dilation. She remained hypotensive, despite high-dose epinephrine and norepinephrine infusions as well as stress-dose hydrocortisone. Admission SARS-CoV-2 IgG assay was positive, meeting the criteria for MIS-C. An ECHO 9 hours after admission demonstrated a severe decline in left ventricular function. Due to severe cardiogenic shock, she was cannulated for venoarterial extracorporeal support (ECMO). During her ECMO course, she was treated with remdesivir, intravenous methylprednisolone, intravenous immunoglobulin, and anakinra. She was decannulated on ECMO day 7, extubated the following day, and discharged home 2 weeks later without respiratory or cardiac support. The use of ECMO for cardiopulmonary support for pediatric patients with MIS-C is feasible and should be considered early as part of the treatment algorithm for patients with severe cardiopulmonary dysfunction.

## Introduction

Severe acute respiratory distress syndrome coronavirus 2 (SARS-CoV-2) causes a severe viral respiratory syndrome known as COVID-19 which has led to significant worldwide morbidity and mortality. Initial published studies suggested that COVID-19 illness might have a milder course in pediatric patients, but severe disease does occur.^1-3^

Additionally, it has been recognized that a small subset of COVID-19-positive pediatric patients have developed an inflammatory syndrome with features similar to Kawasaki disease.^4-6^ This inflammatory syndrome, designated as multisystem inflammatory syndrome in children (MIS-C) in the United States and pediatric inflammatory multisystem syndrome temporarily associated with SARS-CoV-2 (PIMS-TS) in parts of Europe, can manifest as prolonged fever, elevation in laboratory markers of inflammation, and severe illness with multi-organ involvement including cardiac dysfunction, shock, acute respiratory failure, neurologic manifestations, and many others.^4,6,7^ Previous reports have demonstrated high rates of cardiac manifestations including left ventricular depression and coronary aneurysms.^4,7^

Although rare, severe cases of MIS-C have required the use of extracorporeal membrane oxygenation (ECMO) for cardiopulmonary support.^4,5,7^ ECMO provides support for reversible causes of severe cardiac and/or pulmonary failure. Indications for the use of ECMO support continue to expand; however, very little data exist on the management of severe MIS-C with ECMO.

## Case Report

A 5-year-old previously healthy girl presented with 5 days of fever, abdominal pain, sore throat, and dysuria. The day prior to presentation she was seen in an outside hospital emergency department, where streptococcal pharyngitis testing was negative. She was treated for a presumed urinary tract infection with cephalexin and discharged home. The next day she developed a new papular rash and returned to the emergency department with ongoing abdominal pain, nausea, emesis, headaches, and improving dysuria. She had no sick contacts, and no known exposure to SARS-CoV-2. On presentation, she was tachypneic, tachycardic, and hypotensive. SARS-CoV-2 was diagnosed by viral PCR, and a chest radiograph revealed bilateral multifocal pneumonia. She remained tachycardic with worsening hemodynamics, despite fluid resuscitation with 60 mL/kg of .9% saline. She was admitted to the pediatric intensive care unit (PICU) due to acute respiratory failure and fluid-refractory shock. An epinephrine infusion was started for hypotension, high-flow nasal cannula was initiated for acute respiratory failure, and broad-spectrum antibiotics were initiated.

Despite increases in her respiratory support, her breathing and hypoxemia worsened, necessitating intubation. Initial oxygenation index was 19, indicating severe acute respiratory distress syndrome. She was given remdesivir and COVID-19 convalescent plasma. An echocardiogram (ECHO) on admission demonstrated low normal left ventricular function (ejection fraction (EF) 60%) with normal right ventricular function and mild left anterior descending (LAD) coronary artery dilation. She remained hypotensive, despite the addition of stress-dose hydrocortisone and a norepinephrine infusion. SARS-CoV-2 IgG assay testing collected on admission (prior to the administration of COVID-19 convalescent plasma) returned positive, suggesting that this case met the criteria for severe COVID MIS-C, rather than severe acute COVID-19 infection.

Nine hours later, a repeat ECHO demonstrated severely diminished left ventricular function (EF 31%, shortening fraction 14%) with mild LAD coronary artery dilation, and she was requiring high-dose epinephrine and norepinephrine infusions to maintain adequate mean arterial pressures. Oxygenation and ventilation continued to worsen, despite escalating ventilator support. Due to severe cardiogenic shock and myocarditis, venoarterial (VA) ECMO cannulation was performed via right common carotid artery (15-French cannula) and right internal jugular vein (19-French cannula). After cannulation, milrinone was initiated for inotropy, and epinephrine and norepinephrine were weaned as tolerated. High-dose intravenous methylprednisolone, intravenous immunoglobulin (IVIG), and a recombinant interleukin-1 antagonist (rIL1a and anakinra) were given as additional treatment modalities for MIS-C.

On ECMO day (ED) 1, the patient’s epinephrine and norepinephrine infusions were weaned off; however, an ECHO demonstrated persistent LV depression, and rIL1a dose was increased. On ED 2, the patient’s pulse pressure increased, but hypoxemia worsened, and lactatemia developed so ECMO flows were increased from 95 mL/kg/minutes to 120 mL/kg/minutes, with improved oxygenation and resolving lactatemia. On ED 3, the patient became acutely hypotensive, requiring further escalation of ECMO flow to a maximum 150 mL/kg/minutes. Flow was titrated to ensure adequate perfusion. A furosemide infusion was initiated to help with fluid removal and continued until after decannulation. Cardiac function and respiratory status improved while on VA ECMO, and on ED 6, a trial off revealed normal biventricular function, with the ECMO circuit clamped. The patient was decannulated on ED 7. There were no mechanical or hematologic complications, and post-ECMO ECHO showed normal biventricular function.

The patient was extubated to high-flow nasal cannula on post-ED 1 and weaned to room air on post-ED 4. She completed a 5-day course of remdesivir, and her rIL1a and steroids were weaned slowly based on inflammatory markers. She was discharged home on post-ED 15 (hospital day 23), with an oral prednisone taper without cardiac or respiratory support.

## Discussion

We demonstrate the successful use of ECMO to provide cardiorespiratory support for severe MIS-C. This report details one of the youngest patients identified in the literature thus far to require ECMO support secondary to MIS-C. Additionally, in this patient, ECMO was performed without complications, demonstrating feasibility in pediatric patients with MIS-C until their underlying inflammatory syndrome can be reversed.

Previous reports have described the use of ECMO for MIS-C; however, as this is an emerging clinical entity, those reports have been limited to small series and case reports.^7,8^ A report by Riphagen described the therapeutic management and outcomes in 8 patients with MIS-C in South East England. While 7 of 8 patients in this series survived, the patient managed with VA ECMO died, and details of the ECMO course are unclear.^8^ Davies et al described a large, national, multicenter observational study of 78 children admitted to the PICU in the United Kingdom for PIMS-TS. Three of these children required ECMO support. Two of the 78 children died, and details of the patients requiring ECMO were not provided.^5^ Belhadjer et al describe multi-institutional experience with MIS-C in Switzerland and France, which consisted of 35 children, 10 of whom required ECMO support. All 10 of the patients placed on ECMO for MIS-C survived to decannulation, and all, but 1, had been discharged from the hospital at the time of publication.^7^ The largest multicenter observational study of MIS-C was recently published from the United States with 186 patients.^6^ Eight of these patients required ECMO, with 5 surviving to discharge (63%).^6^ In that series, children were treated with intravenous immune globulin, glucocorticoids, interleukin-6 inhibitors, and rIL1a; however, none of the patients from that series received convalescent plasma or remdesivir as these treatment strategies are most common with acute COVID infections.^6^ In all of these varied reports, few details of the ECMO course are given.

In addition to ECMO support, our patient was given inotropic support, diuretic infusion, high-dose corticosteroids, IVIG, COVID-19 convalescent plasma, rIL1a, and remdesivir. There is currently no evidence to which treatments are the most beneficial for MIS-C, and published cases demonstrate the substantial variation in treatment strategies. Riphagen and colleagues^8^ used inotropic support, corticosteroids, and IVIG. While the details of therapies used in the 10 ECMO patients with MIS-C described by Belhadjer are unclear, they do note that of their 35 patients, they treated 28 with inotropic support (80%), 25 with IVIG (71%), 12 with corticosteroids (34%), and 3 with rIL1a (8%). Our therapeutic approach also included a combination of immune modulating agents. Unique to our case was the addition of remdesivir and COVID-19 convalescent plasma as it was initially unclear if this was a case of acute COVID-19 or MIS-C. The treatment of pediatric SARS-CoV-2 patients at our institution with escalating oxygen requirement includes remdesivir as first-line therapy and convalescent plasma, if hypoxemia worsens. However, as the case progressed, it appeared to align more with MIS-C, and additional immune modulators were added. Perhaps, her rapid clinical improvement and full recovery to preadmission health status was related to a combination therapy of several immune modulating agents, as well as brisk cannulation for ECMO to prevent further end-organ dysfunction. Ideally, randomized controlled trials are needed to study the most effective treatment strategies for MIS-C. However, given the rapid advancement of this novel disease, thorough observational reports and detailed success stories such as ours can accumulate evidence for multimodal therapy.

In conclusion, the use of ECMO for cardiopulmonary support for patients with MIS-C is feasible. The use of ECMO was associated with survival to hospital discharge in this case; however, the optimal medical treatment of children with MIS-C is still largely unknown. Using a combination of ECMO, inotropic support, diuresis, COVID-19 convalescent plasma, corticosteroids, IVIG, rIL1a, and remdesivir in a critically ill child with MIS-C demonstrated survival, with return to pre-illness baseline. Further studies are needed to determine the most effective treatment strategy for this novel condition.
